# Inter-laboratory comparison of gene expression biodosimetry for protracted radiation exposures as part of the RENEB and EURADOS WG10 2019 exercise

**DOI:** 10.1038/s41598-021-88403-4

**Published:** 2021-05-07

**Authors:** M. Abend, S. A. Amundson, C. Badie, K. Brzoska, R. Hargitai, R. Kriehuber, S. Schüle, E. Kis, S. A. Ghandhi, K. Lumniczky, S. R. Morton, G. O’Brien, D. Oskamp, P. Ostheim, C. Siebenwirth, I. Shuryak, T. Szatmári, M. Unverricht-Yeboah, E. Ainsbury, C. Bassinet, U. Kulka, U. Oestreicher, Y. Ristic, F. Trompier, A. Wojcik, L. Waldner, M. Port

**Affiliations:** 1 Bundeswehr Institute of Radiobiology Affiliated to University Ulm, Neuherbergstr. 11, 80937 Munich, Germany; 2 Center for Radiological Research, Columbia University Irving Medical Center (CUIMC), New York, USA; 3 Cancer Mechanisms and Biomarkers, Radiation Effects Dept, Centre for Radiation, Chemical and Environmental Hazards, Public Health England Chilton, Didcot, UK; 4 Institute of Nuclear Chemistry and Technology, Centre for Radiobiology and Biological Dosimetry, Warsaw, Poland; 5 Radiation Medicine Unit, National Public Health Center (NPHC), Budapest, Hungary; 6 Department of Safety and Radiation Protection, Forschungszentrum Jülich (FZJ), Jülich, Germany; 7 Centre for Radiation, Chemical and Environmental Hazards, Public Health England Chilton, Didcot, UK; 8 Institute of Radiation Protection and Nuclear Safety, PSE-SANTE/SDOS/LDRI, 92262 Fontenay-aux-Roses, France; 9 Bundesamt für Strahlenschutz (BfS), Federal Office for Radiation Protection, Oberschleissheim, Germany; 10 Centre for Radiation Protection Research, Department of Molecular Biosciences, The Wenner-Gren Institute, Stockholm University, Stockholm, Sweden; 11 Department of Translational Medicine, Medical Radiation Physics, Lund University, Malmö, Sweden

**Keywords:** Genetics, Molecular biology

## Abstract

Large-scale radiation emergency scenarios involving protracted low dose rate radiation exposure (e.g. a hidden radioactive source in a train) necessitate the development of high throughput methods for providing rapid individual dose estimates. During the RENEB (Running the European Network of Biodosimetry) 2019 exercise, four EDTA-blood samples were exposed to an Iridium-192 source (1.36 TBq, Tech-Ops 880 Sentinal) at varying distances and geometries. This resulted in protracted doses ranging between 0.2 and 2.4 Gy using dose rates of 1.5–40 mGy/min and exposure times of 1 or 2.5 h. Blood samples were exposed in thermo bottles that maintained temperatures between 39 and 27.7 °C. After exposure, EDTA-blood samples were transferred into PAXGene tubes to preserve RNA. RNA was isolated in one laboratory and aliquots of four blinded RNA were sent to another five teams for dose estimation based on gene expression changes. Using an X-ray machine, samples for two calibration curves (first: constant dose rate of 8.3 mGy/min and 0.5–8 h varying exposure times; second: varying dose rates of 0.5–8.3 mGy/min and 4 h exposure time) were generated for distribution. Assays were run in each laboratory according to locally established protocols using either a microarray platform (one team) or quantitative real-time PCR (qRT-PCR, five teams). The qRT-PCR measurements were highly reproducible with coefficient of variation below 15% in ≥ 75% of measurements resulting in reported dose estimates ranging between 0 and 0.5 Gy in all samples and in all laboratories. Up to twofold reductions in RNA copy numbers per degree Celsius relative to 37 °C were observed. However, when irradiating independent samples equivalent to the blinded samples but increasing the combined exposure and incubation time to 4 h at 37 °C, expected gene expression changes corresponding to the absorbed doses were observed. Clearly, time and an optimal temperature of 37 °C must be allowed for the biological response to manifest as gene expression changes prior to running the gene expression assay. In conclusion, dose reconstructions based on gene expression measurements are highly reproducible across different techniques, protocols and laboratories. Even a radiation dose of 0.25 Gy protracted over 4 h (1 mGy/min) can be identified. These results demonstrate the importance of the incubation conditions and time span between radiation exposure and measurements of gene expression changes when using this method in a field exercise or real emergency situation.

## Introduction

In a large-scale radiological mass casualty, sensitive and high throughput diagnosis of exposed individuals would be required in order to evaluate the extent of radiation injuries as quick as possible and, when needed, initiate an appropriate treatment^1^. Parameters from physical dosimetry like estimates of the absorbed dose can also help to provide evidence for later occurring acute or chronic health effects. In the absence of physical dosimeters (e.g. in case of terrorist attacks or large-scale nuclear accidents or scenarios when badge dosimeters are not routinely worn by those likely to be exposed), biological changes after radiation exposure can be used for estimation of individual dose. The gold standard in the field of biological dosimetry is scoring dicentric chromosomes. The method is sensitive and very reliable^2^, but it is time consuming and requires several days (lymphocytes in G0 phase have to be stimulated to re-enter the cell cycle where they will be arrested in the metaphase in vitro) before the dose estimates are available^3^. Another emerging technique is based on gene expression analysis observed after radiation exposure. The expression of several genes has already been shown to be modulated in a dose-dependent manner^4–6^ and there is strong evidence for gene expression to be used as an alternative tool for early^7,8^, high-throughput minimally invasive radiation biodosimetry^9–14^ and point of care diagnostic^15^. However, most of this work is based on dose rates in the range of 1000 mGy/min. For decades, the effects of dose rate on clonogenic survival has been very well reported and acknowledged^16,17^. More recently, studies have begun to investigate the effect of dose rate on gene expression in mice^18–20^, human blood and primary cells^21,22^, and occupationally exposed workers^23–26^. In summary, these studies have shown differences from acute exposures with effects of dose, dose-rate, and time since exposure varying by gene.

Many protracted low-dose, low-dose-rate scenarios exist such as environmental contamination and ingestion of radionuclides (e.g. Cs-137) which is a major concern in fallout from a nuclear reactor accident or a terrorist attack^27,28^. There is also concern regarding risk of leukaemia and other tumour entities due to occupational low dose-rate exposure of radiation and medical workers^29,30^. Astronauts on a mission to Mars represent another scenario in which scientists are concerned about haematopoetic responses, cancers, neurological and cardiovascular damage^31^ and for which reliable biological dose and risk estimation is thus highly desirable.

Motivated by these protracted low dose rate scenarios a study was organized in 2019 and performed jointly under the umbrella of the RENEB e.V. network (Running the European Network of Biological dosimetry and physical retrospective dosimetry) together with the EURADOS Working Group (WG) 10 Retrospective Dosimetry. Among other methodologies, gene expression analysis was carried out on four blood samples incubated at 37 °C during exposure to an Iridium-192 source. By varying the distance from the source, dose rates of 2.5–37 mSv/min were achieved and blood samples were exposed over 1 or 2 h. For biodosimetry purposes, gene expression analyses of candidate genes were performed in six independent laboratories using different technologies, namely qRT-PCR and microarrays and different analysis protocols. This study adds to several questions such as the impact of dose rate on gene expression changes performed in different laboratories worldwide as well as the reproducibility and the required degree of protocol harmonization inherent to transcriptomic analysis.

## Materials and methods

### Field exercise: overview

**Figure 1:**
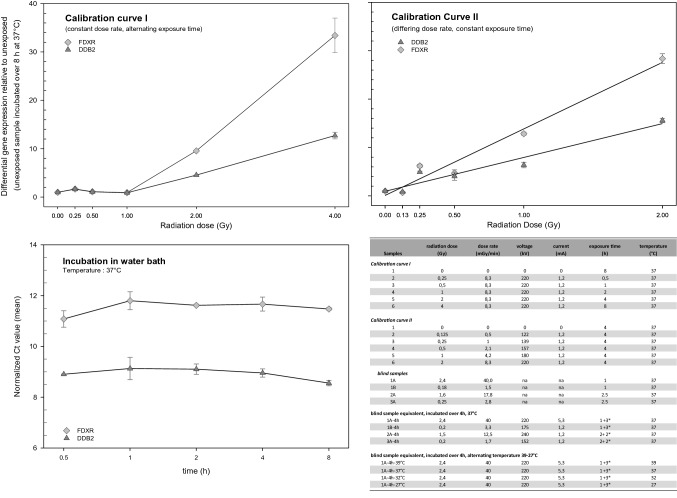
Typical calibration curves I (**A**) and II (**B**) were generated using two radiation-induced genes (*FDXR* and *DDB2*) and exposures as shown in the Table to the bottom right. Unexposed samples incubated over 8 h at 37 °C showed constant gene expression values over time (**C**) indicating that the reference of 0 Gy incubated over 8 h will not introduce a bias in samples irradiated over a shorter period of time as shown in the Table for calibration curve I. Symbols represent mean gene expression values from technical replicates. Error bars represent standard deviation of duplicate measurements and are visible when larger than the symbols. Calibration curves were generated before the exercise (n = 6) and those shown herein represent typical examples (graph created using SigmaPlot Version 14.0, http://www.systatsoftware.com).

All experimental protocols were approved by the central Swedish Ethical Review Authority (Ethical approval: “Dnr 2019-03844”). Aliquots of 5 ml peripheral blood were drawn from a healthy male human volunteer, filled into EDTA-coated vials (Becton Dickinson, Germany) and then stored in thermo bottles filled with preheated water to maintain temperature during exposure. Written informed consent was obtained from all donors. Due to challenging environmental conditions, temperatures in the thermo bottles declined from 37 to 32 °C (1 h exposure) and from 39 to 27.7 °C (in the 2.5 h exposure). Thermo bottles containing EDTA-tubes were attached on three anthropomorphic phantoms and placed at specified distances from an Iridium-192 source (1.36 TBq) to ensure different dose rates and reference doses after irradiation over 1 h and 2 h, as shown in the Table in Fig. 1^32^. After exposure, about 2.5 ml EDTA-blood were transferred into each of two PAXgene Blood RNA tubes (PreAnalytiX GmbH, Qiagen, Hilden, Germany). PAXGene tubes were inverted 10 times and sent by express service at room temperature to the Bundeswehr Institute of Radiobiology (BIR) for RNA isolation and distribution of aliquots of four blinded RNA samples (called 1A, 1B, 2A and 3A) to another five contributing laboratories/teams for dose estimation based on radiation-induced gene expression changes (see Table 1). Institutions and selected assays are as depicted in Table 1. Team numbers mentioned in the text of this manuscript are randomized to anonymize individual laboratory contributions.

**Table 1:** Overview of participating teams, their institutions, and an overview of their contribution for the RENEB 2019 exercise comprising the platform used as well as the genes, calibration samples and further details.

Institution	Platform	# Genes	Gene name	Prerequisites
Cancer Mechanisms and Biomarkers, Radiation Effects Dept, Centre for Radiation, Chemical and Environmental Hazards, Public Health England (PHE), Chilton, UK	qRT-PCR	1	FDXR	Blind samples, calibration curves I and II
Radiation Medicine Unit, National Public Health Center (NPHC), Budapest, Hungary	qRT-PCR	2	FDXR, DDB2	Blind samples, calibration curves I and II, samples were processed by 3 colleagues
Institute of Nuclear Chemistry and Technology (INCT), Warsaw, Poland	qRT-PCR	8	BAX, BBC3, CDKN1A, DDB2, FDXR, GADD45A, GDF15, TNFSF4	Blind samples, calibration curves I and II, additional calibration curve (generated in INCT)
Radiation Biology Unit, Department of Safety and Radiation Protection, Forschungszentrum Jülich GmbH (FZJ), Jülich, Germany	Microarrays	7 resp. 2	TNFSF4, FDXR, DOK7, SPATA18, PHLDA3, LINC00475, VWCE resp. TNSF4, FDXR	Blind samples, inhouse controls, assumption: sample 3A = control
Columbia University Irving Medical Center (CUIMC), New York, NY	qRT-PCR	5	CDKN1A, GDF15, FDXR, DDB2, PCNA	Blind samples, calibration curves I and II
Bundeswehr Institute of Radiobiology (BIR), Munich, Germany	qRT-PCR	2	FDXR, DDB2	Blind samples, calibration curves I and II

### Field exercise irradiation set up

The full description of the irradiation set-up for the blood tubes can be found in Waldner et al.^32^. Two irradiation set-ups were performed and two anthropomorphic phantoms were exposed. Thermos flasks filled with water to maintain blood near 37 °C, were attached on the phantoms and used for gene expression experiments (supplemental 1, chapter 1; supplemental Figure 1 and Table 1). At the same time additional blood samples were exposed under similar conditions and used for dicentric chromosome assay. These results will be published separately. 

### Reference dosimetry for field exercise irradiation

In order to evaluate the doses received by the blood samples, physical dose measurements were performed with small radiophotoluminescent (RPL) glass rods (Φ1.5 × 8.5 mm) made in Ag activated phosphate glass (0.7% Ag in weight). GD 351 type dosimeters supplied by Chiyoda Technol Corporation (Japan) were used. These dosimeters are energy compensated by a tin filter of 0.75 mm and are preferred to the GD301 type without filtration that exhibit an overresponse at low energy due to the non-equivalence of glass to air or tissue: effective atomic number is 12.04. The energy response is almost flat down to 30 keV^33^. The filter also ensures the role of build-up materials ensuring the electronic equilibrium at least up to 4 MV X rays beam^34^. The dose range of applicability is given from 10 µGy to 10 Gy. The reader is a FGD-1000 also from Technol. As RPLs provide a nondestructive reading technique, repetitive measurements of the same dosimeter can be performed. Ten independent measurements were made for each dosimeter, thus providing a good estimation of the reproducibility of measurements. Prior to being sent to Sweden for the field test irradiation, RPL dosimeters were annealed at 400 °C for 30 min to erase the stable Ag0 and Ag2+ color centers before reuse. Prior to measurements, dosimeters were heated at 70 °C for 30 min to stabilize the signal by accelerating the signal build up.

Dosimeters were placed in a sealed vinyl bag for the measurements performed in the container filled with water and on the external surface of the container. Three dosimeters were placed on each blood tube at the surface facing the source along the vertical axis, namely at the top of the tube, the center and the bottom. The blood height in tube was estimated at 6 cm. These dosimeters were placed in this way to evaluate a possible dose gradient on the vertical axis and also to estimate the doses received by the samples. To evaluate a possible problem of position of the water container, four dosimeters were placed in the horizontal plane around the bottle at half height. This also aims to evaluate any possible perturbation caused by the phantom on which the containers were attached.

For the calibration of the RPL signal, RPL dosimeters of the same batch as dosimeters used for the field exercise were irradiated at known dose in a controlled facility. Two types of irradiation were performed to calibrate RPL in terms of absorbed dose in water and air kerma.

The calibration in terms of absorbed dose in water was performed with 4 MV X-rays from LINAC (Elekta) at IRSN, France. RPL irradiated at the LINAC facility were placed in the water tank, with water at 20 °C, according to specification of IAEA TRS-398 protocol^35^. Reference dosimetry was performed with a PTW 31010 ionization chamber calibrated in terms of absorbed dose in water against Co-60 gamma-rays. Dosimeters were also sealed in a vinyl bag. Absorbed dose in water delivered (D*w*) ranged from 0.1 Gy to 3 Gy. Uncertainty on delivered D*w* was estimated at 5% (k = 2).

For calibration of kerma in air, irradiations were performed with the gamma-rays of a Cs-137 radioactive source at the IRSN reference facility. Dosimeters were irradiated in air behind a 2 mm PMMA plate with doses ranging from 10 mGy to 3 Gy. Uncertainty on air kerma was estimated at 2.5% (k = 2).

For these irradiations, two dosimeters were irradiated for each dose and configuration. For the field exercise or for the calibration irradiation, control dosimeters were supplied.

The relative variability of dosimeter response on the batch used was 4.4%. For a few dosimeters, the difference from the batch average value reached 15%. Therefore, a sensitivity correction factor was applied for all dosimeters used in this study to improve the accuracy of the reported doses.

The average signal from control dosimeters was subtracted to get a net signal free from background dose for the different experiments (field test and calibration).

As the temperature of the water filling the containers was 37 °C for the field exercise and 20 °C for the D*w* calibration, the effect of temperature during the irradiation on the RPL signal intensity was estimated. Two sets of six dosimeters were irradiated with 4 MV X-rays in the water tank at a dose of 5 Gy, one in water at 20 °C and the second one at 37 °C.

### X-ray irradiations for calibration curves performed at BIR

**Figure 2:**
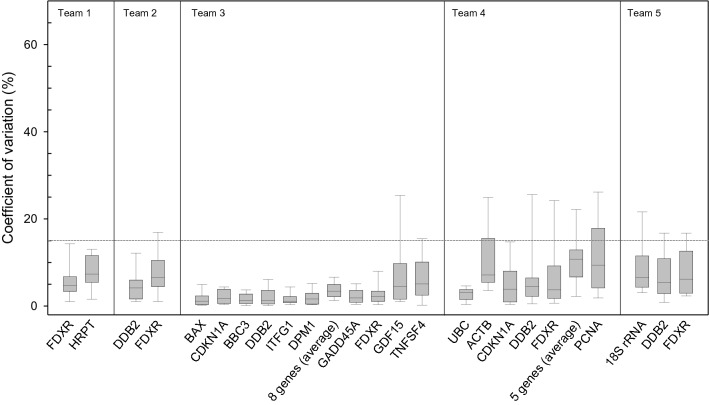
The coefficient of variation (CV) was calculated based on all available technical replicates and genes. The distribution of CVs per gene is reflected by a box plot showing the 10th (lower whisker), 25th (lower end of the box), 50th (median, straight line), 75th (upper end of the box) and 90th (upper whisker) percentiles. Genes are ordered with increasing normalized Ct-values. Teams are arbitrarily numbered from one to five. The horizontal spotted grey line refers to the 15% CV and shows that most measurements are lying below this value (graph created using SigmaPlot Version 14.0, http://www.systatsoftware.com).

Additionally, BIR offered 2 × 6 RNA samples (same donor) so that each laboratory could build two calibration curves when required. In the absence of detailed knowledge of the radiation exposure except that protracted exposure will last over several hours, the first calibration curve was established where blood samples were irradiated at 37 °C using a constant dose rate of 8.3 mGy/min and an exposure time varying between 0.5 and 8 h (Table in Fig. 1). In contrast, EDTA-blood samples for the second calibration curve were exposed to varying dose rates of 0.5–8.3 mGy/min and all blood samples were exposed for 4 h at 37 °C. All the calibration EDTA-blood samples were irradiated submerged in a water bath at 37° C using single doses of X-rays (further details are shown in a corresponding Fig. 2 of chapter 2 of the supplemental 1). X-rays were generated at BIR using a MG325 generator/control unit and an X-ray tube type Y.TU320-D03 (equipped with a 3 mm Beryllium and 3 mm Aluminum filter), which was installed in a Maxishot SPE cabinet (Yxlon, Hamburg, Germany). The absorbed dose was measured using a UNIDOS webline type 10,021 dosimeter using a Farmer chamber TM30010-1 (both from PTW, Freiburg, Germany). The Farmer chamber was calibrated at PTW against Co-60 air kerma and shows a flat response of the correction factor for varying X-ray beam qualities from TH280 to TH70 of about 0.97 with an uncertainty of 2%.

To cover the assumed low dose rates of the iridium irradiation, one steel plate of ~ 5 mm and brass plates of 0.3 mm, 0.5 mm, 1.0 mm and 1.5 mm thickness were placed above the samples and dosimeter during X-Ray irradiation (supplemental 1, chapter 2, supplemental Figure 2). This reduced the dose-rate by a tenth. By additional adjustment of the voltage and the current of the X-Ray tube, dose-rate values were obtained as shown in the Table of Fig. 1.

### Sample processing and analysis

At BIR, RNA was extracted according to the PAXgene Blood miRNA Kit (Qiagen, Hilden, Germany). All methods (e.g. RNA quantity/quality and TaqMan qRT-PCR) were carried out in accordance with the standard operating guidelines, procedures and regulations implemented in our laboratory in 2008 when the Bundeswehr Institute of Radiobiology became DIN-certified by TÜV Süd München, Germany (DIN EN ISO 9001/2008) and were described elsewhere^36,37^. After thawing, washing, and centrifugation of the PAXgene tubes, cells in the supernatant were lysed (Proteinase K, BD Diagnostics, PreAnalytiX GmbH, Hombrechtikon, Switzerland) followed by addition of Lysis/Binding Solution taken from the mirVana Kit (Life Technologies, Darmstadt, Germany). With the mirVana kit, total RNA, including small RNA species, was isolated by combining a Phenol–Chloroform RNA precipitation with further processing using a silica membrane. After several washing steps, DNA residuals were digested on the membrane (RNAse-free DNAse Set, Qiagen, Hilden, Germany), which was then washed. RNA was eluted in water and aliquots were distributed to the teams. Frozen RNA-samples stored on wet ice were shipped by overnight courier service under defined conditions according to United Nations Regulation 650.

Next, RNA was converted into cDNA according to the descriptions in Table 2 for qRT-PCR. Five teams employed qRT-PCR according to protocols established in their laboratories (Table 2). For instance, teams employed different kits for cDNA synthesis, different genes for normalization purposes (e.g. *HPRT* or *18S rRNA*), different radiation-sensitive genes (e.g. *FDXR*, *DDB2*, *BAX*, *GADD45A*) and different amplification protocols (Table 2). For analyses, either normalized threshold cycle (Ct-values) or gene expression fold-changes relative to the unexposed control of the calibration curve were employed. Dose estimates in most laboratories were based on one radio-responsive gene only. Alternatively, in other laboratories, either eight genes were combined (by building the sum of the normalized Ct values) or five genes were combined (by building the geometric mean of the normalized Ct values) and were employed and correlated to the absorbed dose.

**Table 2:** Overview of methodological details of either qRT-PCR or microarrays used by the contributing teams.

Workflow	qRT-PCR				
	PHE	NPHC	INCT	CUMC	BIR
**RNA isolation**					
Isolation kit	n.a	n.a	n.a	n.a	QIAamp RNA Blood Mini Kit
DNA digestion during isolation					RNase-free DNase-Set (Qiagen)
Template eluted in:					RNAse-free water
Quality control					
RNA integrity number					Yes
RNA concentration					Yes (NanoDrop ™)
A260/280					Yes
A260/230					Yes
Check DNA contamination					conventional PCR (ß-actin primer, HotStar MasterMix (Qiagen), 30 cycles)
**cDNA synthesis**					
Kit/MasterMix	High capacity cDNA archive kit	RevertAid First Strand cDNA Synthesis Kit	High Capacity cDNA Reverse Transcription Kit	High-Capacity cDNA Reverse Transcription Kit	High capacity cDNA archive kit
PCR protocol	1×/25 °C/10 min, 1×/37 °C/120 min, 1×/85 °C/5 min	1×/25 °C/5 min, 1×/42 °C/60 min, 1×/70 °C/5 min	1×/25 °C/10 min, 1×/37 °C/120 min, 1×/85 °C/5 min	1×/25 °C/10 min, 1×/37 °C/120 min	1×/25 °C/10 min, 1×/37 °C/120 min
Quality control	HPRT1 Ct	18S rRNA Ct	ITFG1 Ct, DPM1 Ct	XXX	18S rRNA Ct
**qRT-PCR**					
Kit/MasterMix	TaqMan,PerfeCTa®, MultiPlex qPCR SuperMix, Quanta bioscience	TaqMan fast advanced master mix	TaqMan Universal Master Mix II	TaqMan Universal Master Mix	TaqMan Universal Master Mix
TaqMan assay	FDXR, no. HS01031617_m1	DDB2 (Hs00172068_m1), FDXR (HS01031617_m1), 18SrRNA (Hs99999901_s1)	BAX (Hs00180269_m1), BBC3 (Hs00248075_m1), CDKN1A (Hs00355782_m1), DDB2 (Hs03044953_m1), FDXR (Hs00244586_m1), GADD45A (Hs00169255_m1), GDF15 (Hs00171132_m1), TNFSF4 (Hs00182411_m1), ITFG1 (Hs00229263_m1), DPM1 (Hs00187270_m1)	CDKN1A (Hs00355782_m1), DDB2 (Hs03044953_m1), FDXR (Hs00244586_m1), GDF15 (Hs00171132_m1), PCNA (Hs00696862_m1)	DDB2 (Hs00172068_m1), FDXR (HS01031617_m1)
Cycles	1×/95 °C/2 min, 40×/95 °C/10 s, 60 °C/1 min	1×/50 °C/2 min, 1×/95 °C/20 s, 40×/95 °C/3 s, 60 °C/30 s	1×/95 °C/10 min, 40×/95 °C/15 s, 60 °C/1 min	1×/50 °C/2 min, 95 °C/10 min, 40×/95 °C/15 s, 60 °C/1 min	1×/50 °C/2 min, 1x/95 °C/10 min, 40×/95 °C/1 min, 60 °C/1 min
Detection system	Rotor Gene Q (Qiagen)	Rotor-Gene Q (Qiagen)	7500 Real-Time PCR System (Thermo Fischer Scientific)	QIA-7	GeneAmp 7900
Threshold	fixed	fixed	fixed	0.05	automatic
Normalization	HPRT1	18S rRNA	ITFG1 and DPM1 (geometric mean)	UBC	Human 18S rRNA
Quantification method	D-Ct approach	DD-Ct method, relative fold change in relation to the 0 Gy sample of the Calibration curve	D-Ct approach	Relative Quantification	D-Ct approach
Quality control					
Standard curve	Yes	No	No	NA	Yes
Slope	Yes	No	No	NA	Yes
r2	Yes	No	No	NA	Yes
18s rRNA Ct	HPRT1, (–)RT and NTC	18S rRNA Ct, NTC	ITFG1, DPM1, NTC	NA	Yes

Microarrays					
Microarray	Agilent, 44 k whole human genome, G4112F				
RNA amount used for cDNA synthesis	0.4 µg; 1.65 µg per array				
cDNA amount used for cRNA synthesis	N.D				
Number of interrogated genes	> 41,000 transcripts				
Software	Agilent Feature Extraction Software (v.9.5.1); Agilent GeneSpring GX software				
Set normalisation algorithm	Processed signal value were log2-transformed, median normalized				

The sequence of topics from top to bottom reflect a typical workflow for gene expression analysis starting with the isolation of RNA, quality and quantity controls, cDNA synthesis and qRT-PCR.

Calibration curves were used separately by most participants, thus, several dose estimates were generated (e.g. two dose estimates were reported when using *FDXR* and calibration curves I and II). Alternatively, calibration curves (delivered calibration curves I and II as well as internal laboratory-specific calibration curves) were combined (building the sum of the normalized Ct values) aggregating the number of dose estimates. Finally, different software tools such as Statistica 9.0 software (StatSoft), GraphPad Prism, version 6.04 (GraphPad Software, La Jolla, CA, USA), R 3.6.2 or Sigma Plot 14 (Jandel Scientific, Erkrath, Germany) were employed to fit the calibration curves and to perform dose estimates.

Gene expression analysis using DNA microarrays was performed on the Agilent platform as described elsewhere^11^. The mRNA (400 ng of total RNA per sample) was transcribed into cDNA with an oligo-dT primer, followed by transcription into cRNA labeled with cyanine 3-CTP (Quick-Amp Labeling Kit, One-color, Agilent). cRNA purification was performed with the RNeasy Mini Kit (Qiagen) and dye incorporation and cRNA yields were measured with the NanoDrop-1000 spectrophotometer. Labeled cRNA samples were applied on the DNA microarray slides (44 k whole human genome, G4112F, Agilent). For hybridization, DNA microarrays were placed into a hybridization oven (44 K Agilent) at 65 °C for 17 h. After hybridization, DNA microarrays were washed and slides were immediately scanned with the Microarray Scanner (G2505 B, Agilent) as recommended by Agilent. The pre-processing procedure and subsequent statistical analysis were applied separately using Agilent Feature Extraction Software Version 9.5.1 (Agilent processed signal value) and Agilent GeneSpring GX software. By initial data filtering control features and non-uniform outliers were excluded, as well as signals that were not significantly above the background intensity in 25% of all samples. Remaining signals were subsequently log2-transformed and median normalized. Dose estimation was performed using a 7-gene signature as well as a 2-gene signature using internal calibration curves^12,13,38^.

Further details on each method are provided in the supplement (supplemental word datafile 1).

### Statistical methods

Descriptive statistics were calculated in Excel. Graphs were created using SigmaPlot Version 14.0 (Jandel Scientific, Erkrath, Germany). Linear or linear-quadratic curve fits were performed where applicable (SigmaPlot, Version 14.0). The Supplemental File 1 includes more statistical details for each team where required.

## Results

### Field dosimetry and true dose estimates

 For the field test, higher dose heterogeneity was observed for the tubes exposed the closest to the source (during the first irradiation) reaching 15%, whereas for the longer distance, it did not exceed 3%. The heterogeneity is due to geometry of the irradiation. This fact is confirmed also by the RPL dosimeters placed on the thermoflask. For the thermos flask with sample P1A, a very large vertical dose gradient was observed on the front side of the flask, from 0.34 Gy at of the bottom flask to 2.48 Gy at the flask top. A difference as also observed between the left and right side of the flasks, respectively 2.33 to 1.74 Gy. These data show the importance of measuring the dose directly on the tube, also taking into account the difficulty of accurately positioning the tubes in the flask. The reference dose provided for each tube was the average of the doses estimated from the three dosimeters on each tube. The dose heterogeneity is taken into account in the uncertainty estimation. The temperature effect on RPL dosimeters (20 °C vs 37 °C) was found to be less than the reading reproducibility (about 3%) and therefore was considered here as non-significant.

Hence, true (reference)-dose estimates were 2.38 ± 0.37 Gy (k = 1) for sample 1A, 1.56 ± 0.15 Gy (k = 1) for sample 2A, 0.18 ± 0.03 Gy (k = 1) for sample 1B and 0.25 ± 0.01 Gy (k = 1) for sample 3A, respectively (Table 3).

**Table 3:** The reported dose estimates from teams running qRT-PCR or microarrays are shown for each blinded sample irradiated with a known (true) dose, which is shown in parenthesis in the subtitles.

Teams	Calibration curves used	Method	Reported dose estimates (Gy)			
			1A (true dose, 2.4 Gy)	1B (true dose, 0.18 Gy)	2A (true dose, 1.6 Gy)	3A (true dose, 0.25 Gy)
1	FDXR and calibration curve I	qRT-PCR	0.13	0.05	0.15	0.04
	FDXR and calibration curve II		0.04	0.02	0.05	0.02
2	FDXR and calibration curve I, 3 individuals measured	qRT-PCR	0.11	0.13	0.14	0.09
	DDB2 and calibration curve I, 3 individuals measured		0.29	0.34	0.41	0.33
	FDXR and calibration curve II, 3 individuals measured		< 0.125	< 0.125	< 0.125	< 0.125
	DDB2 and calibration curve II, 3 individuals measured		< 0.125	< 0.125	< 0.125	< 0.125
3	8 signature genes + three calbration curves	qRT-PCR	0.09	0.04	0.13	0.01
4	7-genes, inhouse calibration curves; assumption: sample 3A = control	microarrays	0.25	0.8	0.25	0
	2-genes, inhouse calibration curves; assumption: sample 3A = control		0.1	0.2	0.05	0
5	5 signature genes + both calibration curves used	qRT-PCR	0.3	0.2	0.3	0.2
6	FDXR and calibration curve I	qRT-PCR	0	0	0	0
	FDXR and calibration curve II		0–0.2	0	0	0
	DDB2 and calibration curve I		0.63	0	0	0
	DDB2 and calibration curve II		0.22	0	0.17	0

Use of different calibration curves and genes generates several dose estimates per team.

### Quality/quantity of calibration and blinded samples and candidate genes

The quality checks performed at BIR before distributing RNA aliquots indicated successful (DNA free) RNA isolation of sufficient amounts of high-quality RNA (mean RIN of 9.0, SEM ± 0.18) from all blood samples (≥ 6 µg total RNA, except for one sample from which 4.5 µg total RNA was isolated). Hence, all laboratories received 1 µg (0.75 µg for one sample) total RNA.

### Methodological precision of qRT-PCR

We examined the precision of qRT-PCR gene expression measurements by performing technical replicates in duplicate/triplicates. In the case of microarrays, no replicate measurements of calibration samples were performed. A coefficient of variation (CV, standard deviation relative to the mean RNA copy number) of ≤ 10% and a CV of ≤ 15% was found in 85.1% and 92.6% of altogether 650 qRT-PCR measurements (Fig. 2). Lower CVs (≤ 5%) were observed for 10 genes by team#3 and overall CVs increased with higher raw Ct-values, which is reflected by larger box-plots to the right of each laboratory, since the order of genes per team in Fig. 2 follows the increasing Ct-value.

### Number of participating institutions, contributions and reported dose estimates

Six participating institutions (Table 1) provided 16 contributions and a total of 64 dose estimates (Table 3). For instance, team#1 examined a single gene (*FDXR)* using both calibration curves^38,39^. This represents two contributions, generates two dose estimates per blinded sample and a total of 8 dose estimates (Table 3). Team#2 used two different genes (*FDXR* and *DDB2*) both with two calibration curves separately. Averaged gene expression values were converted into four corresponding dose estimates per blinded sample and a total of 16 dose estimates were reported. Team#4 using microarrays estimated the dose based on linear-quadratic equations on a previously identified gene set consisting of 7 genes and a reduced 2-gene signature. No further calibration samples were required. Instead, internal calibration curves of the 7 signature genes were used. The derived log_2_ values of the 7 signature genes in some of the received RNA samples were far lower than the values of the non-irradiated controls of the internal calibration curves. Among them, sample 3A showed the overall lowest log_2_ values and was, therefore, selected to be a non-irradiated sample and log_2_ values were normalized to the internal non-irradiated control. Based on this, the log_2_ values of all other samples were re-scaled. After re-scaling, the log_2_ values were used to derive dose estimates based on the internal calibration curves. The two contributions (7-gene signature and a reduced 2-gene signature) resulted in two dose estimates per blinded sample and a total of 8 dose estimates (Table 3).

### Calibration curves and reported versus true dose estimates

Before the start of this exercise calibration curves (data not shown) were generated at BIR and an example was distributed to the participants along with RNA samples to build their own calibration curves (Fig. 1). For improved comparison, differential gene expression was calculated relative to the unexposed samples incubated over 8 h (calibration curve I) and 4 h (calibration curve II). Figure 1C, shows constant expression of *FDXR* and *DDB2* throughout the 8 h incubation time at 37 °C indicating that the reference 0 Gy sample with 8 h incubation time (calibration curve I) can be used without introducing a bias in samples irradiated over a shorter period of time as shown in the Table for calibration curve I in Fig. 1. Both calibration curves revealed a threshold, and increased gene expression of *FDXR* and *DDB2* was only observed at doses exceeding 1 Gy (calibration curve I) and 0.125 Gy (calibration curve II) and exposure times > 2 h (Fig. 2 and Table in Fig. 1).

**Figure 3:**
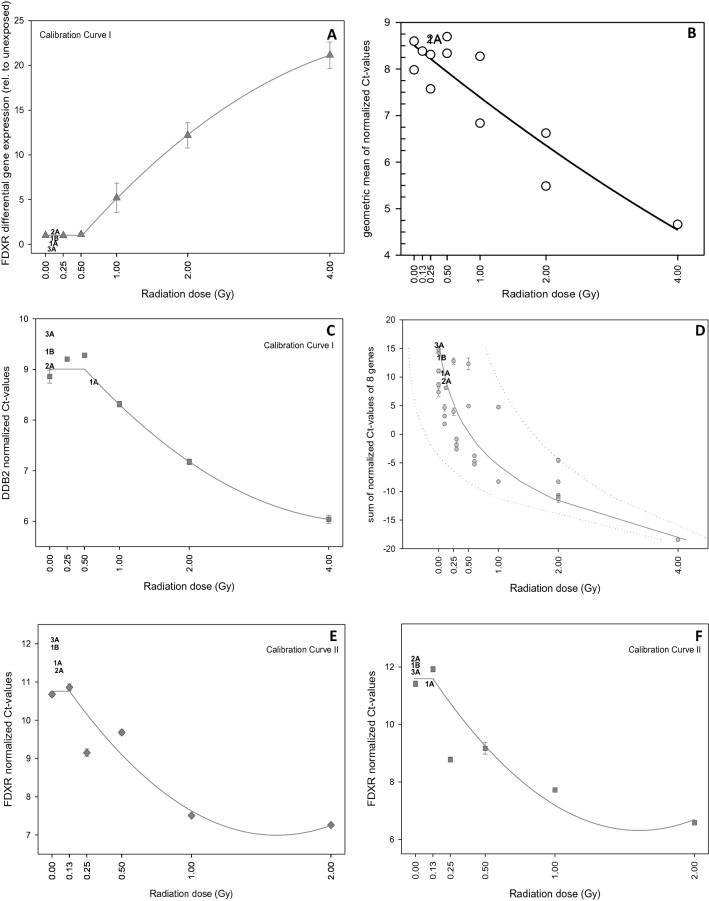
Examples of calibration curves I (**A**,**C**) and II (**E**,**F**) and additional calibration curves combined (**B**,**D**) generated by the different qRT-PCR teams are shown. Gene expression reflected either as differential gene expression (reference was the 0 Gy calibration sample, **A**), normalized single genes from two different teams (**C**,**E**,**F**), averaged (**B**) or summed Ct-values (**D**) using several genes combined were plotted versus radiation dose (Gy). Normalized and differential gene expression values measured below 0.5–1.0 Gy (calibration curve I) and 0.125 Gy (calibration curve II) were fitted with a horizontal line (assuming a threshold) and higher values using a manual fit. For summed normalized Ct-values of 8 genes (*BAX, BBC3, CDKN1A, DDB2, FDXR, GADD45A, GDF15, TNFSF4*) and utilizing different calibration curves an exponential fit was used and the 95% confidence intervals were plotted using a dotted line. As an example a linear-quadratic fit was used in (**B**) (details are presented in Supplemental File 1). Error bars represent standard deviation from duplicate measurements except for (**A**), where duplicate measurements were performed by each of three different individuals. Error bars are visible when larger than the symbols. Blinded samples 1A, 1B, 2A and 3A corresponding to the team’s calibration curve are superimposed in the panels and sometimes overlapping each other, because of very similar values (graph created using SigmaPlot Version 14.0, http://www.systatsoftware.com).

Contributing teams plotted either differential gene expression, single, summed or averaged normalized Ct-values versus radiation dose (Fig. 3). The different teams confirmed the above-mentioned threshold in both calibration curves, below which no changes in gene expression after exposures up to 0.5–1.0 Gy (calibration curve I) or 0.125 Gy (calibration curve II) were detected. Blinded samples in most cases revealed Ct-values or fold-gene expression changes falling in the dose band showing no association or saturation with gene expression (Fig. 3).

Reported dose estimates across all teams and different contributions on average revealed about 10-times lower dose estimates for the higher true-dose samples 1A (reported average dose of 0.2 Gy versus true dose of 2.38 Gy) and 2A (reported average dose of 0.15 Gy versus true dose of 1.56 Gy, Table 3). True doses in the 0.2 Gy range resulted in estimates within the same order of magnitude. Thus, for sample 1B, the average reported dose was 0.15 Gy versus the true dose of 0.18 Gy, and for sample 3A, the average reported dose was 0.07 Gy versus the true dose of 0.25 Gy (Table 3).

**Figure 4:**
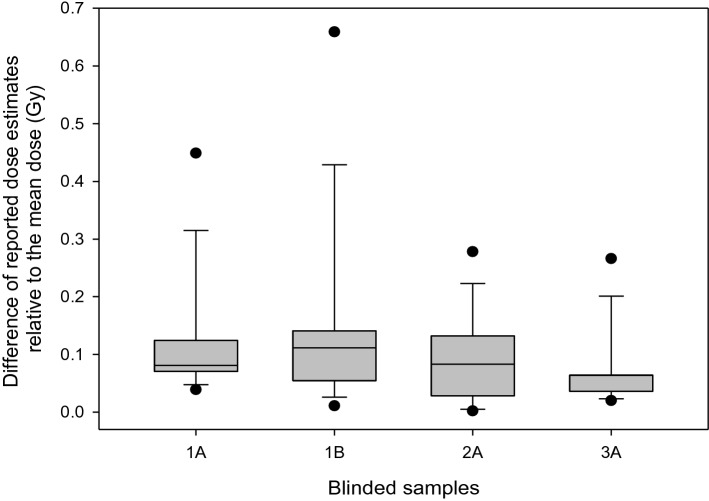
The box plot reflects the absolute difference of all reported dose estimates from all teams relative to the mean reported dose value per blind sample. The box plot shows the 10th (lower whisker), 25th (lower end of the box), 50th (median, straight line), 75th (upper end of the box) and 90th (upper whisker) percentiles (graph created using SigmaPlot Version 14.0, http://www.systatsoftware.com).

Inter-laboratory comparison revealed consistent dose estimates over all contributions, and the reported dose estimates differed in 75% of the data by ≤ 0.14 Gy (Fig. 4).

**Figure 5:**
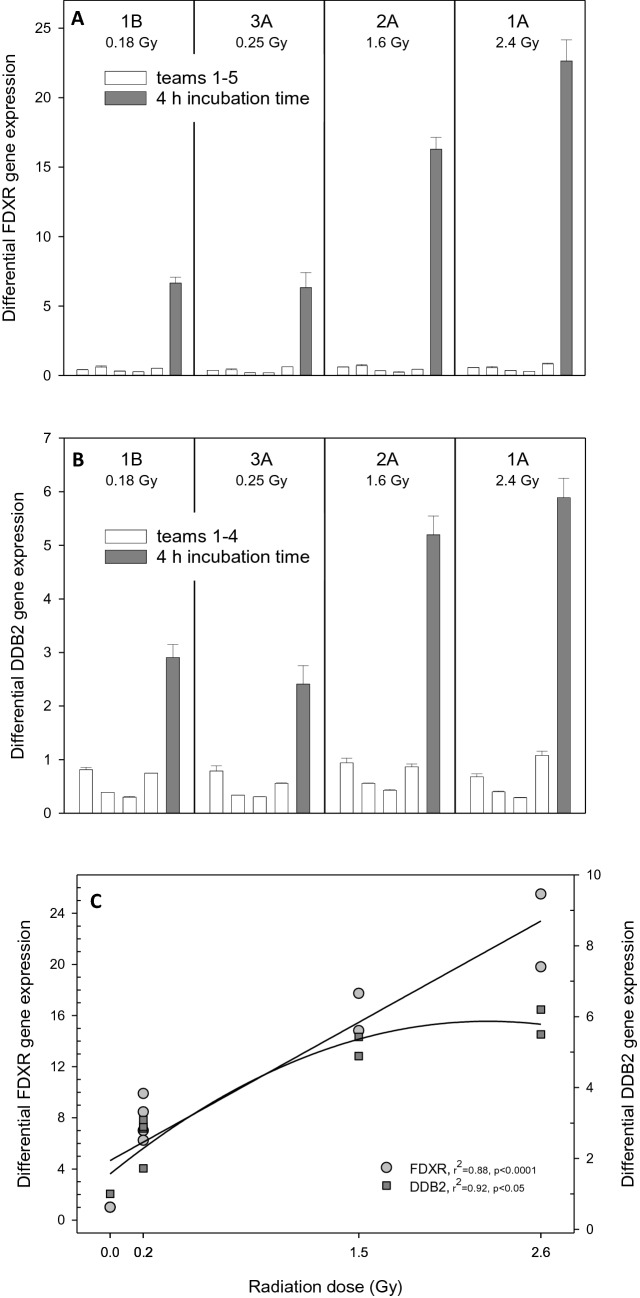
Differential gene expression changes of the blinded samples for each team were calculated relative to the 0 Gy samples of the calibration curve I for inter-comparison purposes. White bars represent the fold-changes for the genes used for dose estimation of the blinded samples (1A, 1B, 2A and 3A) by different teams arbitrarily numbered 1–5 (**A**) and 1–4 (**B**). Exposure time was 1 h and 2 h for the blinded samples with no additional time allowed for gene expression to respond. When using blinded samples with an adjusted exposure and incubation time of 4 h combined, much larger gene expression changes were observed (dark grey bar), more in line with previous reports at these doses. Results are shown for *FDXR* (**A**) and *DDB2* (**B**). The observed changes in gene expression follow a linear fit of *FDXR* and a linear-quadratic fit of *DDB2* with absorbed dose (**C**). Error bars represent the standard error of the mean and bars/dots the mean from replicate measurements (graph created using SigmaPlot Version 14.0, http://www.systatsoftware.com).

Differential gene expression changes in the blinded samples were calculated from each team’s *FDXR* and *DDB2* measurements relative to the 0 Gy samples of calibration curve I. This produced values of 0.20–0.84 (*FDXR*) and 0.29–1.1 (*DDB2*), which is in the magnitude of the unexposed controls (Fig. 5). In order to assess the impact of post-exposure expression time on relative gene expression levels, additional samples were irradiated at BIR laboratory using the same parameters of exposure as the blinded samples of the field exercise, but allowing a total combined exposure and incubation time of 4 h (Table in Fig. 1). Under these conditions, the expected linear (*FDXR)* and linear-quadratic (*DDB2)* dose dependent gene expression changes were observed (Fig. 5).

**Figure 6:**
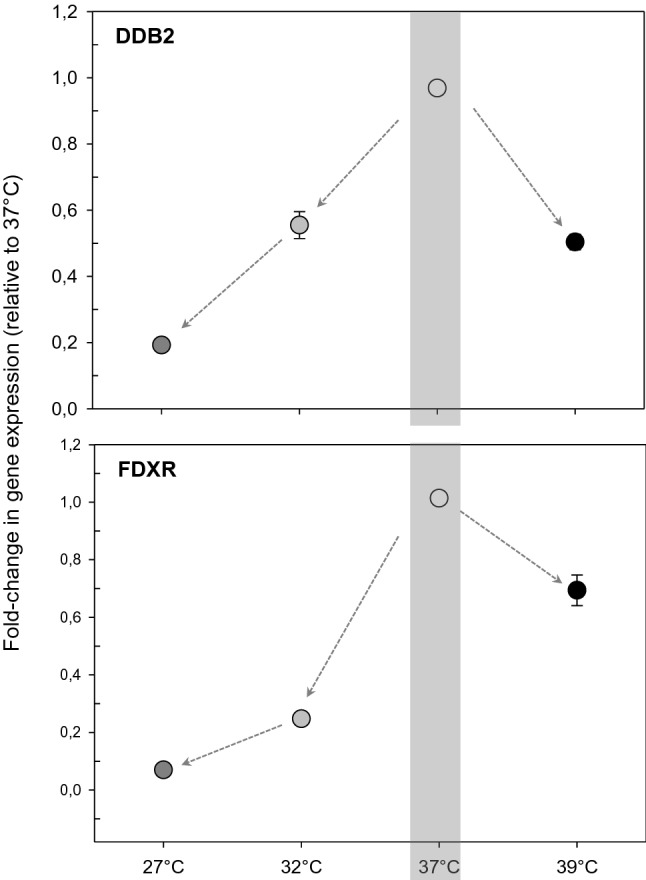
Changes in differential gene expression (DEG) of *DDB2* (upper graph) and *FDXR* (lower graph) relative to 37° C (calibrator) were examined at a range of temperatures between 27 and 39 °C with an additional 3 h incubation time at corresponding temperatures. Symbols are average gene expression fold changes and error bars are SEM (standard error of the mean) from two independent experiments on two blood samples with two technical replicates (total n = 8) (graph created using SigmaPlot Version 14.0, http://www.systatsoftware.com).

Additional experiments were performed to assess the effect of temperature on relative gene expression of *FDXR* and *DDB2* reflecting a range between shipping and body temperatures. We tested reduced (32 °C or 27 °C) and increased (39 °C) temperatures during an exposure of 2.4 Gy (40 mGy/min) over 1 h plus an additional 3 h incubation time. We observed that deviations from 37 °C decreased *FDXR* and *DDB2* copy number between 0.25 and onefold per degree Celsius relative to 37 °C (Fig. 6).

## Discussion

Potential large scale protracted low dose and low dose-rate scenarios are omnipresent and include environmental contamination and ingestion of radionuclides via fallout from a nuclear reactor accident or after a terrorist attack, occupational exposure of radiation and medical workers or planned human exploration missions to Mars^25,27–31,40^. Our current RENEB and EURADOS WG 10 study reflects a range of doses and dose-rates because blood samples were exposed to an Iridium-192 source at varying distances, resulting in low dose rates of 1.4–43.5 mGy/min with exposures lasting over 1 or 2 h (Fig. 1). Dose estimates available that early after irradiation allow for an early hospitalization as well as early treatment e.g., early administered countermeasures like cytokines^41^, which will have a high impact on the outcome of acute health effects. For biodosimetry purposes, gene expression analyses of candidate genes were applied in six different laboratories using qRT-PCR and microarrays and employing different protocols. Teams did choose the genes they felt comfortable with. For instance, previous examinations comparing in vivo and in vitro blood models in baboons and humans (leucemia patients) provided strong evidence for the applicability of e.g. *FDXR* or *DDB2 *in vitro^42^.

As expected, qRT-PCR methodology was characterized by high precision reflected by a low coefficient of variation of replicate measurements examined over all teams, which is in agreement with previous findings using this technology^43^. This highlights the advantage of gene expression measurements for biodosimetry purposes, as different protocols were utilized (Table 2). It also points to the required degree of harmonization for qRT-PCR methodology, which appears to be lower compared to the application of the dicentric chromosomal assay, except that certain quality criteria (e.g. RNA quality) as outlined by the MIQE statements have to be considered^44,45^. As a limitation, we have to acknowledge a reduction in methodological variance caused by RNA isolation of all blood samples by one group and the distribution of RNA-aliquots to the other laboratories. In addition, inter-individual variance was not considered, since blood samples originated from one donor only.

Reported dose estimates from all teams were generated based on gene expression data lying mostly outside the range (below a threshold) where a significant dose-to-gene association was observed (Fig. 3). This occurred most likely because the time during/after irradiation was too short to allow for substantial gene expression changes to happen. Since these results were reported by all six teams using different techniques and protocols, this highlights the method’s ability to reproducibly inform about detection limits. Also, dose estimates among teams showed low variance (≤ 0.14 Gy), underscoring the consistency of the method across different laboratories (Fig. 4).

From calibration curve I, it becomes evident that given a constant dose rate of 8.3 mGy/min, a 4-h exposure time is required for a significant and greater than twofold change in gene expression for e.g. *FDXR* and *DDB2* (Fig. 1A). However, in calibration curve II, corresponding significant gene expression changes were seen at a total dose of only 0.25 Gy delivered at a rate of 1 mGy/min (Fig. 1B). As this exposure required 4 h, significantly longer than the exposure times in calibration curve I, and post-exposure incubation time is known to be important in gene expression studies, we considered that the total exposure time may have contributed to the relative non-responsiveness observed in the blind samples. Because the blind samples were exposed using higher dose rates, the 1–2 h exposure times may have been too short to allow radiation-induced changes in gene expression to occur.

That conclusion is in agreement with published data. For instance, radiation-induced gene expression changes have been reported to occur at 2–6 h (*FDXR*) and 3–8 h (*DDB2*) after a single exposure with dose rates around 1 Gy/min in lymphocyte or whole blood cultures in vitro and in whole blood drawn from patients^9,12,46–50^. Furthermore, large-scale murine studies inform about gene expression changes detected at dose rates even below 1 mGy/min, but in this case the mice were exposed over almost 500 days and the cumulative doses were 0.02–8 Gy^51^. This was found in another mouse study as well^30^. Further in vitro studies showing radiation-induced gene expression changes applying dose rates of about 3 mGy/min and higher and exposures lasting over several hours, augment the interpretation of our data so that the total exposure and expression time of 1 and 2 h appears to have been insufficient to allow the biological response of the transcriptome^52–54^.

As a test of this interpretation, we irradiated samples in the same way as the blinded samples, but added a post-irradiation incubation to increase the total incubation time (including the exposure time) to 4 h. We observed expected gene expression changes in these samples, which showed a significant association with the absorbed dose (Fig. 5, grey bars). Clearly, a total exposure and incubation time of 4 h was required to allow for early changes in gene expression, making this a prerequisite for running the gene expression assay. This is comparable to a 2 h post-exposure incubation time at 37 °C used in the in vitro dicentric chromosome assay to allow DNA repair after radiation exposure. This is consistent with our observation that a protracted dose of 0.25 Gy applied at 1 mGy/min over 4 h can be detected based on the genes and the model applied (Fig. 1). Radiation-induced gene expression changes over time, thus, adding to the complexity of this approach^55^. However, recently published work in this regard indicates time-windows (up to 3 days), where genes such as *FDXR* or *DDB2* can be used for biodosimetry purposes^50^. Also, when using gene expression changes for biodosimetry purposes, we are measuring a biological response of the organism and not e.g. DNA-strand breaks as indicated by the dicentric assay. Deciphering the underlying impacted biological processes is challenging, but gene´s annotation and bioinformatic analysis provide some clues in this regard.

It should also be noted that all of the teams had developed their biodosimetry models and selected the genes exclusively using acute radiation exposures (high dose rate), although the transcriptomic response is known to vary with different dose rates^18–21^. In this context we experienced higher than expected variance in gene expression of *FDXR* and *DDB2* in additional experiments for establishing the calibration curves, which might be caused by the low dose-rates used in these experiments. Future studies might identify different gene sets as more appropriate for different exposure situations (e.g. low versus high dose rate). Nevertheless, the findings from our 4-h study suggest that our biodosimetry models may have utility for dose reconstruction of low dose-rate exposures if the time element can be satisfactorily accounted for. The possibility of incorporating genes with unique responses to low dose-rate irradiation may provide a means to distinguish between acute and low dose-rate exposures, or to improve dose estimates in cases of low dose-rate exposure. Such information could be important in situations where mixed exposures are possible, as protraction of radiation exposure is known to modify the resulting biological effects on important endpoints such as cell survival^14,16^ and haematopoiesis^31,56–58^. In future studies it will be important to determine the extent to which reduced estimates of biological dose, relative to acute exposure models or physical dose, correspond to the actual biological injury sustained.

Some teams employed an unexposed reference for dose estimation, but other teams used normalized gene expression changes, exemplifying that dose estimates can be generated even without a pre-exposure control (Fig. 3). Employing this assay for dose estimation even in the absence of a pre-exposure control is another desirable feature of gene expression measurements for dose estimation and is in agreement with previous findings^9^.

Our results highlight the requirement for sustained temperature control during and after exposure, which was compared with temperature effects on cytogenetic measurements performed in a field exercise. We conclude that gene expression appears more sensitive to temperature in comparison with cytogenetic endpoints. Further work is underway to compare the results of the gene expression parts of this study with the dicentric assay and other dosimetry methods reported in Waldner et al.^30^.

Regarding the “lessons learned” from this exercise, going forward it will be important to clearly define the requirements for each assay and for key players from all teams to discuss a detailed draft plan prior to future inter-comparison exercises. In the current study, experimental parameters that were sub-optimal for gene expression studies led to challenges such as a low environmental temperature during irradiation, and the subsequent need to perform additional control experiments. However, analysis of these experiments also extended our understanding of the impact of temperature on gene expression, as well as aiding interpretation of the main study. In addition to the general agreement of dose estimates across the participating laboratories, a key finding of this exercise was that gene expression signatures selected to provide dose reconstruction of acute exposures may need to be modified for use when low dose-rate exposure is suspected. Further experimental work will be needed to determine if the reduced response of these genes correlates well with the reduced biological damage inflicted by protracted doses, or if selection of specific low dose-rate reporter genes is required for use in such a scenario.

In conclusion, gene expression measurements are (1) highly reproducible and are (2) very precise even when using different techniques, protocols and laboratories. (3) The required degree of harmonization is low and (4) no pre-exposure control is required. (5) Even protracted radiation of 0.25 Gy over 4 h with 1 mGy/min can be identified, provided that the time required for the biological response (at least 4 h, including the exposure time in our study) as well as a constant temperature of 37 °C were considered. This makes radiation-induced transcriptional changes an attractive method for biodosimetry purposes and demonstrates the importance of both the time span between radiation exposure and measurements of gene expression changes when using this method in a field exercise or real emergency situation.

## Supplementary Information

Supplementary Information.
