# Supplementary material for: Inter-laboratory comparison of gene expression biodosimetry for protracted radiation exposures as part of the RENEB and EURADOS WG10 2019 exercise
Source: Sci Rep. 2021 May 7;11:9756. doi: 10.1038/s41598-021-88403-4 (PMC8105310; doi:10.1038/s41598-021-88403-4)
Supplement: Supplementary file 1 — Supplementary Information. [file 41598_2021_88403_MOESM1_ESM.docx]

**Inter-laboratory comparison of gene expression biodosimetry for protracted radiation exposures as part of the RENEB and EURADOS WG10 2019 exercise**

M. Abend^1*^, S. A. Amundson^2*^, C. Badie^3*^, K. Brzoska^4*^, R. Hargitai^5*^, R. Kriehuber^6*^, S. Schüle^1^, E. Kis^5^, S. A. Ghandhi^2^, K. Lumniczky^5^, S. R. Morton^2^, G. O´Brien^3*^, D. Oskamp^6^, P. Ostheim^1^, C. Siebenwirth^1^, I. Shuryak^2^, T. Szatmári^5^, M. Unverricht-Yeboah^6^, E. Ainsbury^7^, C. Bassinet^8^ U. Kulka^9^, U. Oestreicher^9^, Y. Ristic^8^, F. Trompier^8^, A. Wojcik^10^, L. Waldner^11^, M. Port^1^

^1^Bundeswehr Institute of Radiobiology, Munich, Germany;

^2^Center for Radiological Research, Columbia University Irving Medical Center (CUIMC), New York, USA;

^3^Cancer Mechanisms and Biomarkers, Radiation Effects Dept, Centre for Radiation, Chemical and Environmental Hazards, Public Health England Chilton, United Kingdom;

^4^Institute of Nuclear Chemistry and Technology, Centre for Radiobiology and Biological Dosimetry, Warsaw, Poland;

^5^Radiation Medicine Unit, National Public Health Center (NPHC), Budapest, Hungary;

^6^Department of Safety and Radiation Protection, Forschungszentrum Jülich (FZJ), Jülich, Germany;

^7^Centre for Radiation, Chemical and Environmental Hazards, Public Health England Chilton, United Kingdom;

^8^ Institute of Radiation Protection and Nuclear Safety, PSE-SANTE/SDOS/LDRI, 92262 Fontenay-aux-Roses, France;

^9^Bundesamt für Strahlenschutz (BfS), Federal Office for Radiation Protection, Oberschleissheim, Germany;

^10^Centre for Radiation Protection Research, Department of Molecular Biosciences, The Wenner-Gren Institute, Stockholm University, Stockholm, Sweden.

^11^Lund University, Department of Translational Medicine, Medical Radiation Physics, Malmö, Sweden

**Key words**

Protracted ionizing radiation, gene expression, biodosimetry, inter-laboratory comparison, microarray, TaqMan, qRT-PCR

**Running title**

Third RENEB/EURADOS gene expression study

**1^st^ revised Version**

submitted February 2021

to

**Scientific Reports**

**Figures:** 6

**Tables:** 3

**Supplemental word data file:** 1

Corresponding Author:

Michael Abend, M.D.

Bundeswehr Institute of Radiobiology affiliated to University Ulm

Neuherbergstr. 11, 80937 Munich, Germany

Tel.: +49-89-992-692-2280

FAX: +49-89-992-692-2255

e-mail: michaelabend@bundeswehr.org

*these authors contributed equally

**Supplemental 1**

Abend et al.

1. **Reference dosimetry for the field exercise**


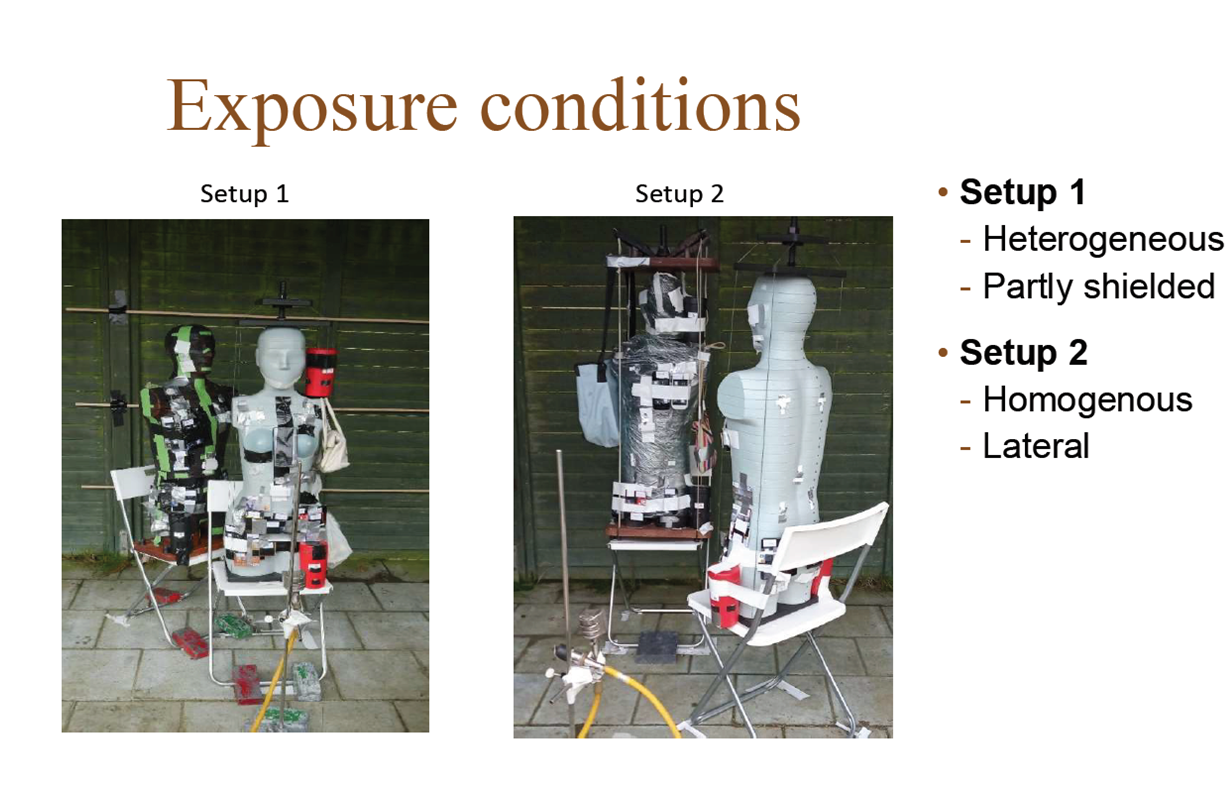


**Supplemental figure 1;** Pictures of the set-up of the two irradiations performed during the field test.

| **Tube coding** | **Irradiation number** | **Phantom code** | **Phantom position** | **Position on phantom** |
| --- | --- | --- | --- | --- |
| 1A | 1 | Phantom 1 | Antero posterior | Front, left, bottom |
| 1B | 1 | Phantom 1 | Antero posterior | Left side of the phantom head |
| 2A | 1 | Phantom 2 | Facing but with 45° angle | Front, right, bottom |
| 3A | 2 | Phantom 3 | Lateral Left | Left lateral side |

**Supplemental table 2;** the table provides the positioning of the different blood tubes on the different phantoms for the two irradiations.

1. **Graphical presentation of the dosimetry for the low dose rate *in vitro* exposure at Bundeswehr Institute of Radiobiology (BIR)**


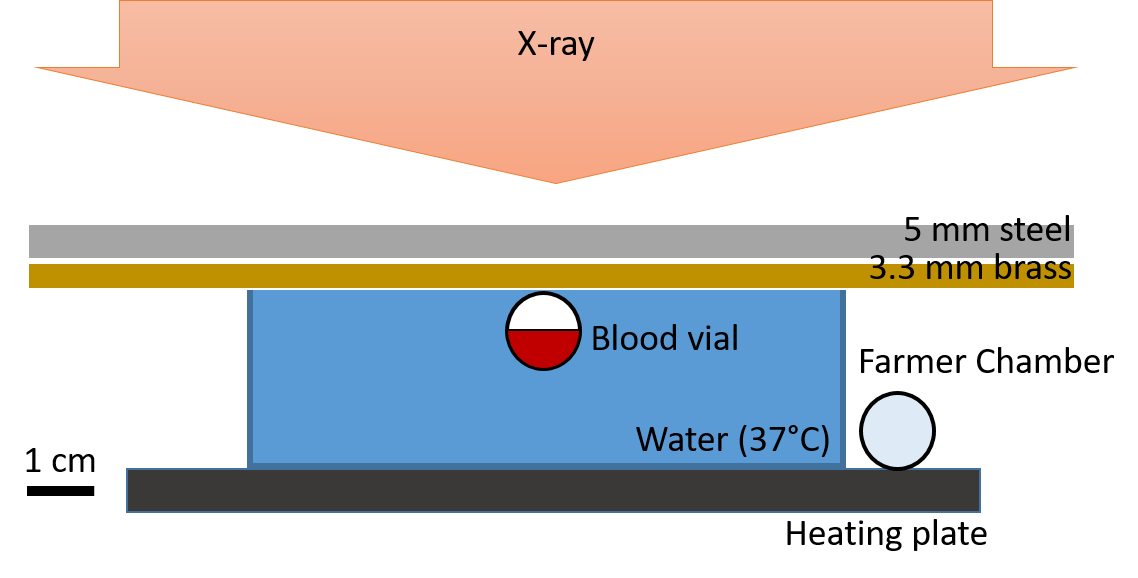


**Supplemental figure 2;** Description of the low dose-rate exposure setting for blood irradiation *in vitro* at Bundeswehr Institute of Radiobiology.

1. **Details on methods and statistics of contributing laboratories where required**

**3.1 Institute of Nuclear Chemistry and Technology (INCT), Warsaw, Poland**

Calibration curve and dose prediction:

Calibration curve was generated using three groups of samples:

1) Samples from calibration curve generated in INCT. This group of samples includes blood from three healthy donors X-irradiated (acute doses: 0, 0.1, 0.3, 0.6, 2 Gy) and incubated in 37°C for 6 h.

2) Samples from calibration curve I obtained from BIR.

3) Samples from calibration curve II obtained from BIR.

Samples from the above groups were pulled together and one calibration curve was generated based on the sum of ΔCt values of the radiation responsive genes under study (*BAX*, *BBC3*, *CDKN1A*, *DDB2*, *FDXR*, *GADD45A*, *GDF15*, *TNFSF4*). The Statistica 9.0 software (StatSoft) was then used to compute the relationship between sum of ΔCts and radiation dose. The following exponential regression model was calculated an used to predict the radiation dose absorbed by the unknown samples:

$$y=-0.11804+exp(-0.45187-0.10289\times x)$$

Where:

*y* – dose (Gy)

*x*- sum of ΔCt values of *BAX, BBC3, CDKN1A, DDB2, FDXR, GADD45A, GDF15, TNFSF4*

**3.2 Radiation Biology Unit, Department of Safety and Radiation Protection, Forschungszentrum Jülich GmbH (FZJ), Jülich, Germany**

A 7-gene signature (*FDXR, VWCE, TNFSF4, PHLDA3, LGR6, DOK7, SPATA18*) and a therefrom derived reduced 2-gene signature (*FDXR, TNFSF4*) was used to assess radiation doses in this study, based on our previous experience and published literature (11,12,31). Gene expression analysis using DNA microarrays was performed on the Agilent platform. Obtained RNA was processed with Thermo Scientific GeneJET RNA Cleanup and Concentration Micro Kit (Thermo Fisher Scientific Inc., U.S.) due to the given protocol by the manufacturer to meet the requirements for the A260/230 value to be processed properly on the used microarray platform. The mRNA (400 ng of total RNA per sample) was transcribed into cDNA with an oligo-dT primer, followed by transcription into cRNA labeled with cyanine 3-CTP (Quick-Amp Labeling Kit, One-color, Agilent). cRNA purification was performed with the RNeasy Mini Kit (Qiagen) and dye incorporation and cRNA yields were measured with the NanoDrop-1000 spectrophotometer. Labeled cRNA samples were applied on the DNA microarray slides (44k whole human genome, G4112F, Agilent). For hybridization, DNA microarrays were placed into a hybridization oven (Agilent) at 65°C for 17 h. After hybridization, DNA microarrays were washed and slides were immediately scanned with the Microarray Scanner (G2505 B, Agilent) as recommended by Agilent. The pre-processing procedure and subsequent statistical analysis were applied separately using Agilent Feature Extraction Software Version 9.5.1 (Agilent processed signal value) and Agilent GeneSpring GX software. By initial data filtering control features and non-uniform outliers were excluded, as well as signals that were not significantly above the background intensity of 25% of all samples. Remaining signals were subsequently log_2_-transformed and median normalized. Dose estimation were performed using a 7-gene signature as well as a 2-gene signature using internal calibration curves (11,12,31). No further calibration samples were required. Instead, internal calibration curves of the 7 signature genes were used. The derived log_2_ values of the 7 signature genes in some of the received RNA-sample were far lower than the values of the non-irradiated controls of the internal “in house” calibration curves. Among them, sample 3A showed the overall lowest log_2_ values and was, therefore, selected to be a non-irradiated sample and log_2_ values were normalized to the internal non-irradiated control. Based on this, the log_2_ values of all other samples were re-scaled. After re-scaling, the log_2_ values were used to derive dose estimates based on the internal calibration curves. The two contributions (7 gene signature and a reduced 2 gene signature) resulted in two dose estimates per blinded samples and a total of 8 dose estimates.

**3.3 Columbia University Irving Medical Center (CUIMC), New York, NY**

We selected five strongly radiation-responsive genes (*CDKN1A, GDF15, FDXR, DDB2* and *PCNA*) and one housekeeping gene (*UBC*) for this analysis, based on our previous experience and the published literature (20). Since all of the selected radiation-responsive genes have similar dose response shapes with a positive correlation with dose of acute irradiation, using each one as an independent predictor could result in strong multicollinearity in the model. Therefore, we combined them into a single predictor variable by calculating the geometric mean of their log-2 transformed signals on each of the 12 provided samples with known radiation doses. For normalization, the signal values for the housekeeping gene were subtracted from the geometric mean of radiation-responsive gene signals to generate the predictor, called net_sig.

We then built a simple mathematical model (calibration curve) to relate net_sig to the known radiation dose. Visual inspection of the data suggested an exponential function with the following equation, where *D* is radiation dose, *k*_1_ and *k*_2_ are adjustable parameters:

$D=k_{1}\times exp[-k_{2}\times net\_sig]$ (1)

This simple structure with only 2 parameters was selected because the data set of known doses was very small (only 12 samples), and complicated models with many parameters would be likely to strongly overfit the limited data.

To evaluate the performance and stability of our model, we randomly split the known dose data into training and testing halves (6 samples each), fitted the model (Eq. 1) to the training data, and assessed the coefficient of determination (R^2^) and root mean squared error (RMSE) for model predictions on the testing data. This training/testing procedure was repeated 1000 times. Model fitting was performed by a robust nonlinear least squares algorithm, implemented by the *nlrob* function in *R* 3.6.2 software. The model showed stable behaviours over the random data splits. The best-fit mean values for the parameters across the 1000 random training data sets were: *k*_1_ = 99.5 (standard deviation, SD = 53.7), *k*_2_ = 0.67 (SD = 0.11). Mean R^2^ was 0.86 across all training and testing data, and mean RMSE was 0.37 Gy on training data and 0.47 Gy on testing data, respectively.

For comparison, we tried the same procedures on somewhat different gene subsets (e.g. dropping some of the 5 radiation-responsive genes and/or adding 1-2 additional housekeeping genes) and different model versions (e.g. linear instead of exponential). However, the original choice of genes (5 radiation-responsive, 1 housekeeping) and model (Eq. 1) showed the best results in terms of R^2^ and RMSE values and stability on training/testing splits.

Consequently, this original choice of genes and model was fitted to the full known dose data set, which produced best-fit *k*_1_ = 89.0 (standard error, SE = 47.2), *k*_2_ = 0.67 (SE = 0.10), which are consistent with values obtained on splitting the data. The best-fit plot (calibration curve) is shown as graph B in figure 3, where circles indicate the sample values and the curve indicates the model fit.

The best-fit model (Eq. 1) was used to predict the radiation dose on the 4 samples with unknown doses, which are reported here. The unknown samples had net_sig values ranging from 8.6 to 9.2, mean = 8.9. Such values correspond to low doses (<1 Gy) on our calibration curve, leading to model predictions in this range.
